# The density of macrophages in the invasive front is inversely correlated to liver metastasis in colon cancer

**DOI:** 10.1186/1479-5876-8-13

**Published:** 2010-02-08

**Authors:** Qiang Zhou, Rui-Qing Peng, Xiao-Jun Wu, Qing Xia, Jing-Hui Hou, Ya Ding, Qi-Ming Zhou, Xing Zhang, Zhi-Zhong Pang, De-Sen Wan, Yi-Xin Zeng, Xiao-Shi Zhang

**Affiliations:** 1 State Key Laboratory of Oncology in South China, Cancer Center, Sun Yat-Sen University, 651 Dongfeng R E, 510060, Guangzhou, China; 2 Biotherapy Center, Cancer Center, Sun Yat-Sen University, 651 Dongfeng R E, 510060, Guangzhou, China; 3 Department of Colorectal Oncology, Cancer Center, Sun Yat-Sen University, 651 Dongfeng R E, 510060, Guangzhou, China; 4 Department of Pathology, Cancer Center, Sun Yat-Sen University, 651 Dongfeng R E, 510060, Guangzhou, China

## Abstract

**Background:**

Although an abundance of evidence has indicated that tumor-associated macrophages (TAMs) are associated with a favorable prognosis in patients with colon cancer, it is still unknown how TAMs exert a protective effect. This study examined whether TAMs are involved in hepatic metastasis of colon cancer.

**Materials and methods:**

One hundred and sixty cases of pathologically-confirmed specimens were obtained from colon carcinoma patients with TNM stage IIIB and IV between January 1997 and July 2004 at the Cancer Center of Sun Yat-Sen University. The density of macrophages in the invasive front (CD68TF_Hotspot_) was scored with an immunohistochemical assay. The relationship between the CD68TF_Hotspot _and the clinicopathologic parameters, the potential of hepatic metastasis, and the 5-year survival rate were analyzed.

**Results:**

TAMs were associated with the incidence of hepatic metastasis and the 5-year survival rate in patients with colon cancers. Both univariate and multivariate analyses revealed that the CD68TF_Hotspot _was independently prognostic of survival. A higher 5-year survival rate among patients with stage IIIB after radical resection occurred in patients with a higher macrophage infiltration in the invasive front (81.0%) than in those with a lower macrophage infiltration (48.6%). Most importantly, the CD68TF_Hotspot _was associated with both the potential of hepatic metastasis and the interval between colon resection and the occurrence of hepatic metastasis.

**Conclusion:**

This study showed evidence that TAMs infiltrated in the invasive front are associated with improvement in both hepatic metastasis and overall survival in colon cancer, implying that TAMs have protective potential in colon cancers and might serve as a novel therapeutic target.

## Background

Colorectal cancer is the fourth leading cause of cancer deaths worldwide. Of patients with colorectal cancer, 35%-55% will develop hepatic metastases at some time during the course of their disease. Survival following hepatic resection of colorectal metastasis now approaches 35%-50%. However, approximately 65% of patients will have a recurrence at 5 years. Identifying the markers for hepatic metastasis would be helpful for the early treatment of patients at high-risk of hepatic metastasis [1-5].

In addition to clonal selection and the predetermined metastatic potential of cancer cells, there is increasing evidence indicating that the microenvironment modifies the metastasis of cancer cells [6-9]. Cancer tissue is infiltrated with stromal cells including macrophages. Tumor-associated macrophages (TAMs) are not only abundant in epithelial cancers, but also involved in cancer progression [10-13]. Experimental data have indicated that ablation of macrophage function or inhibition of macrophage infiltration into experimental tumors inhibits tumor growth and metastases [14]. Additionally, gene array studies of diagnostic lymph node specimens in follicular lymphoma have shown that genes associated with a strong 'macrophage' signature are associated with a poorer prognosis, independent of clinical variables or of gene expression of the tumor cells [15]. Therefore, TAMs might promote tumor progression by induction of chronic inflammation, matrix remodeling, tumor invasion, intravasation, angiogenesis, and seeding at distant sites [13]. In contrast, recruitment of TAMs also contributes to the development of an adaptive immune response against cancer. TAMs contribute to the balance between antigen availability and clearance through phagocytosis and subsequent degradation of senescent or apoptotic cells. The role of TAMs is essential for triggering, instructing, and terminating the adaptive immune response [16]. The clinical evidence regarding the relationship between TAMs and tumor progression is tumor type-dependent. The higher density of TAMs is associated with a poorer prognosis in leiomyosarcomas, melanomas, gliomas, and cancers of the breast, bladder, rectum, and endometrium, but the prognosis is favorable in nasopharyngeal, gastric, and ovarian cancers [17-28]. Additionally, in liver, lung, and prostate cancers, the role of TAMs on prognosis is controversial [29-35].

With respect to colorectal carcinomas, clinical data indicate that TAMs are associated with a favorable prognosis [36-39]. However, these studies have not indicated the sites at which TAMs show a protective effect. Because macrophages modify tumor invasion, intravasation, and angiogenesis, whether or not TAMs interfere with hepatic metastasis of colon cancer was determined in the current study.

## Materials and methods

### Materials

One hundred and sixty cases of pathologically-confirmed specimens were obtained from colon carcinoma patients with TNM stage IIIB and IV between January 1997 and July 2004 at the Cancer Center of Sun Yat-Sen University. Patients with stage IV colon carcinoma who were enrolled in this study had primary colon cancer with synchronous liver metastasis, irrespective of extra-hepatic involvement. Ninety-eight patients with stage IIIB colon carcinoma underwent radical surgery, while 62 patients with stage IV colon carcinoma underwent palliative colon resection with or without resection of hepatic lesions. None of the patients had undergone either chemotherapy or radiotherapy before the collection of the samples. The histopathologic characteristics of the colon carcinoma tissue specimens were confirmed by blinded review of the original pathology slides. The TNM classification system of the UICC (edition 6) was used for clinical staging, and the World Health Organization classification was used for pathologic grading. The study was conducted in accordance with the Helsinki Declaration and approved by the Ethics Committee of our institution. Patients were informed of the investigational nature of the study and provided their written informed consent.

### Follow-up of stage IIIB patients and post-operative treatment

Clinical follow-up was only provided to stage IIIB patients, as patients with stage IV in this study were a group with high heterogeneity, including solitary or multiple liver metastases, and liver only or other sites involved with metastases; these variables affected the treatment protocols and eventually the response rate and prognosis. Ninety-eight patients with stage IIIB colon carcinoma were observed on an every-3-month basis during the 1^st ^year, once every 6 months in the 2^nd ^year, and by telephone or mail communication once every year thereafter for a total of 5 years. If recurrence or metastasis occurred, 5-FU-based chemotherapy was administered according to the NCCN guidelines [40]. Overall survival (OS) was defined as the time from surgery to death, or was censored at the last known alive data. Liver metastasis-free survival (LMFS) was defined as the time from surgery to liver metastasis.

### Immunohistochemistry

The specimens were fixed in formaldehyde and embedded in paraffin. Only blocks containing the tumor front were evaluated. Tissue sections of 5-μm thickness were cut, dried, deparaffinized, and rehydrated in a series of alcohols and xylene before antigen retrieval by pressure cooker treatment in citrate buffer (pH 6.0) for 3 minutes. After that, we performed endogenous peroxidase blocking through hydrogen peroxide incubation. Mouse anti-human CD68 monoclonal antibody (mAb) (PG-M1; DakoCytomation, Glostrup, Denmark) at a 1:300 dilution was used. Immunostaining for CD68 was performed using EnVision + Dual Link Kit (Dako Cytomation) according to the manufacturer's instructions. The development was performed with a substrate-chromogen solution (3,3'-diaminobenzidine dihydrochloride [DAB]) for 3-5 minutes (brown reaction product). Sections were then counterstained with hematoxylin and mounted in non-aqueous mounting medium.

To analyze macrophage phenotypes, antibodies were stained as follows: 1) IL-12 mAb (1:30, catalog number: sc-74147, mouse IgG1, Santa Cruz biotechnology, CA, USA), 2) human leukocyte antigen (HLA)-DR mAb (1:300, catalog number: ZM-0136, mouse IgG2b, Zhongshan Goldenbridge biotechnology, Beijing, China), 3) IL-10 Ab (1:400, ab34843, rabbit polyclonal Ab, Abcam), 4) transforming growth factor beta1 (TGF-β1) mAb (1:800, catalog number: sc-146, rabbit IgG, Santa Cruz biotechnology, CA, USA).

### CD68 evaluation

Referring to Forssell's [36] scoring system, CD68 immunostaining along the tumor front was evaluated over the whole section (7-10 fields per section) and tumors containing small areas among which the infiltration of CD68-positive cells was considerably above the average level of CD68-positive cells was defined as CD68 hotspots (CD68TF_Hotspot_) [36]. All sections were evaluated far from necrosis areas and H.E. staining was reviewed in case of uncertainty. The CD68TF_Hotspot _of the two highest view fields measured at ×200 magnification was semi-quantitatively graded as no/weak (grade 1), moderate (grade 2), strong/robust (grade 3), and massive infiltration (grade 4). Tumors classified as 1 included completely negative specimens, as well as specimens containing some scattered CD68-positive cells along the tumor margin. Tumors were classified as 2 when CD68 staining was continuous along the tumor margin, but was not extended from the tumor front more than one cell layer on average. CD68 staining that, on average, extended 2-3 cell layers from the tumor margin over the whole section was classified as 3, whereas to be classified as 4, CD68 staining extended several cell layers from the tumor margin in all fields. Each section was scored independently by two independent observers. Interobserver agreements for the CD68TF_Hotspot _were 81%. Disagreements were re-evaluated until a consensus decision was made.

### Statistical analysis

The relationship between the various clinicopathologic characteristics and the CD68TF_Hotspot _parameters were compared and analyzed using χ^2 ^tests, likelihood ratio, and linear-by-linear association, as appropriate. The cumulative survival time was computed using the Kaplan-Meier method and compared by the log-rank test. Univariate and multivariate analyses were based on the Cox proportional hazards regression model. A two-tailed P < 0.05 was considered to be statistically significant. All statistical analyses were performed using SPSS 13.0 software for Windows (SPSS Inc., Chicago, IL, USA).

## Results

### CD68 expression

**Figure 1:**
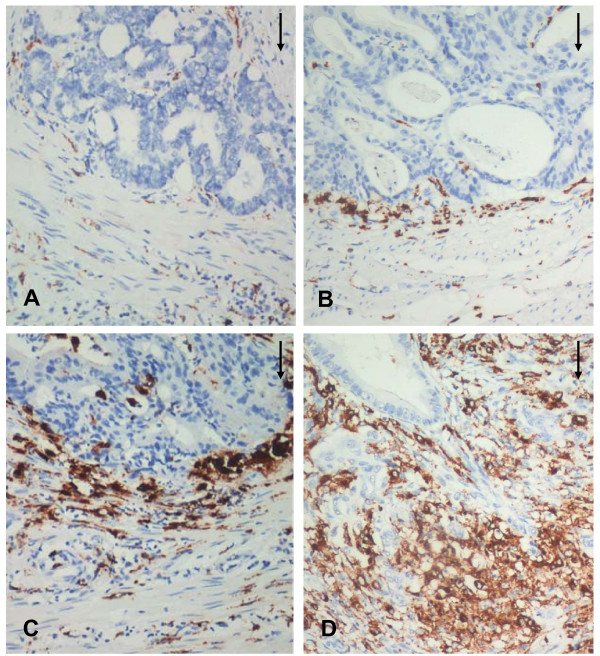
**Representative pictures of CD68TF_Hotspot _in colon cancer patients (200× magnification)**. Different grades of macrophage infiltration along the tumor front were examined with immunohistochemical assay: A, no/low, B, moderate, C, high, and D, massive. Arrows point at tumor front.

TAMs were stained brown in the cytoplasm. The majority of CD68-positive cells were located in the stroma, and in particular, along the invasive front. CD68-positive cells were mostly in apparent direct contact with or immediately adjacent to tumor cells lining the invasive front. Although most areas along the invasive front displayed a fairly homogeneous CD68+ infiltration pattern, there were also tumors containing small areas that showed CD68 infiltration considerably above the average grade (CD68TF_Hotspot_). The CD68TF_Hotspot _was semi-quantitatively graded from 1-4 (Fig. 1).

**Figure 2:**
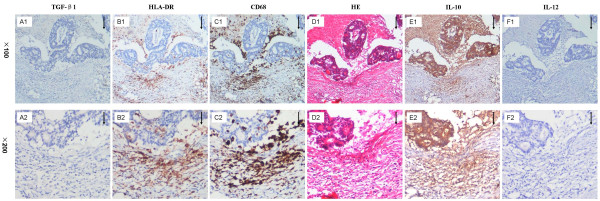
**Representative images of macrophage phenotypes in colon cancer on consecutive sections**. Arrows point at tumor front.

To identify the phenotype of TAMs, a group of consecutive sections was used to stain with CD68, HLA-DR, TGF-β1, IL-10, and IL-12. TAMs were popularly stained with HLA-DR, IL-10, sporadically stained with TGF-β1, negatively stained with IL-12, indicating that TAMs were activated without classic M1 or M2 phenotype (Fig. 2).

### Relationship between CD68TF_Hotspot _and clinicopathologic characteristics

We used the χ^2 ^test to assess the relationship between the TAMs and clinicopathologic characteristics. The results showed that the CD68TF_Hotspot _was inversely correlated with TNM stage, the presence of hepatic metastasis, and pathologic classification (Table 1). When hepatic metastasis status was cut into the following three patterns, the CD68TF_Hotspot _was also highly correlated with the status of hepatic metastasis: no hepatic metastasis (stage IIIB colon cancer without liver metastasis within 5 years of follow-up), metachronous hepatic metastasis (stage IIIB colon cancer with liver metastasis within 5 years of follow-up), and synchronous liver metastasis (stage IV colon cancer with liver metastasis before palliative surgery).

**Table 1:** Correlation between CD68TF_Hotspot _and clinicopathologic characteristics.

Variable	CD68TF_Hotspot_				P value
		
	-/+	+	++	+++	
		
	1	2	3	4	
Gender					
Male	23	21	37	13	0.939
Female	15	13	27	11	
Age (years)					
< 60	22	17	26	15	0.195
≥ 60	16	17	38	9	
Sites of primary tumors					
Left	25	14	40	16	0.107
Right	13	20	24	8	
TNM stages					
IIIB	17	18	46	17	0.025*
IV	21	16	18	7	
Invasive depth					
T3	31	30	53	17	0.422^a^
T4	7	4	11	7	
Hepatic metastasis(1)					
No	13	14	42	16	0.004*
Yes	25	20	22	8	
Hepatic metastasis(2)					
No	13	14	42	16	0.001*^b^
Metachronous	4	4	4	1	
Synchronous	21	16	18	7	
Grade					
G1	1	1	1	0	0.124^b^
G2	23	21	48	21	
G3	14	11	14	2	
G4	0	1	1	1	
Pathologic classification					
Papillary + tubular	28	25	57	23	0.022*^a^
Mucoid + signet ring	10	9	7	1	
Growth pattern					
Pushing	19	8	18	8	0.071
Infiltrating	19	26	46	16	

*: p < 0.05. a: Likelihood ratio. b: Exact linear-by-linear association test.

### Survival analyses

By the end of the 5-year follow-up, 68 of patients with stage IIIB colon carcinoma were alive, thus the 5-year survival rate was 69.4%. Based on univariate analysis, including all stage IIIB patients applicable to survival analyses (n = 98), age, gender, tumor invasive depth, pathologic grade, and growth pattern showed no prognostic significance for OS and LMFS (Table 2). In contrast, the sites of primary tumors, pathologic classification, and hepatic metastasis were predictors for OS. The CD68TF_Hotspot _was highly correlated to OS (*P *= 0.001; log rank test; data not shown), but not LMFS (*P *= 0.221; log rank test; data not shown).

**Table 2:** Univariate analyses of factors associated with OS and LMFS.

Variable	OS (n = 98)		LMFS (n = 98)	
	
	HR, (95% CI)	P value	HR, (95% CI)	P value
Gender (female vs. male)	1.157 (0.562-2.381)	0.693	0.416 (0.114-1.510)	0.182

Age (< 60 y vs. ≥ 60 y)	0.732 (0.352-1.519)	0.402	0.704 (0.230-2.153)	0.538

Invasive depth (T4 vs. T3)	1.023 (0.392-2.674)	0.962	0.902 (0.200-4.068)	0.893

Sites of primary tumors (right vs. left)	2.271 (1.093-4.717)	0.028*	0.815 (0.267-2.491)	0.720

Grade (G3 vs. G2 vs. G1)	1.519 (0.715-3.224)	0.277	1.036 (0.324-3.311)	0.953

Pathologic classification (mucoid + signet ring vs. papillary + tubular)	2.415 (1.129-5.168)	0.023*	1.148 (0.316-4.171)	0.834

Growth pattern (infiltrating vs. pushing)	0.817 (0.389-1.718)	0.595	2.709 (0.600-12.223)	0.195

CD68TF_Hotspot _(4 vs. 3 vs. 2 vs.1)	0.568 (0.393-0.822)	0.003*	0.594 (0.344-1.025)	0.061

CD68TF_Hotspot _group (high vs. low)	0.288 (0.139-0.600)	0.001*	0.324 (0.106-0.991)	0.048*

Hepatic metastasis (yes vs. no)	5.852 (2.737-12.511)	0.000**	NA	NA

Univariate analysis, Cox proportional hazards regression model. Abbreviations: HR, hazard ratio; CI, confidence interval; NA, not assessment. *: p < 0.05; **: p < 0.001.

**Figure 3:**
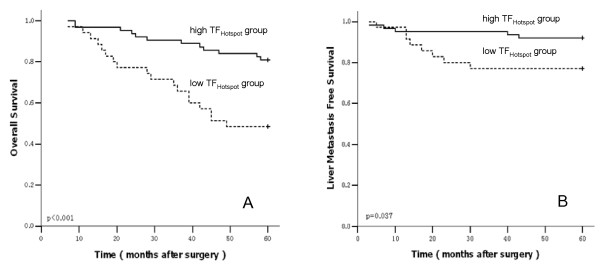
**Kaplan--Meier analysis of overall survival (A) and liver metastasis-free survival (B) for CD68TF_Hotspot _group**. The patients with a higher CD68TF_Hotspot _group (solid lines) were associated with longer 5-year overall survival and liver metastasis-free survival than those with a lower CD68TF_Hotspot _group (dashed lines).

For further analysis, the grade data of the CD68TF_Hotspot _were divided into 2 groups (grade 1 and 2 versus 3 and 4) according to Forssell's protocol [36]. Therefore, cases were regrouped into CD68TF_Hotspot _high (3 and 4) versus CD68TF_Hotspot _low (1 and 2) macrophage infiltration. Kaplan-Meier survival curves were then plotted to further investigate the association with OS. The log-rank statistic was used to compare survival rates. There was a positive association between the CD68TF_Hotspot _group and both OS (P < 0.001) and LMFS (P = 0.037; Fig. 3).

### Multivariate Cox proportional hazards analysis

Whether or not the CD68TF_Hotspot _group could serve as an independent predictor of OS and LMFS was analyzed. A multivariate Cox proportional hazards analysis was performed, including gender, age, sites of primary tumors, invasive depth, grade, pathologic classifications, liver metastasis, growth patterns, and CD68TF_Hotspot _groups. In stage IIIB colon cancers, the high CD68TF_Hotspot _group had a significantly lower risk for OS (hazard ratio [HR], 0.433; 95% confidence interval [CI], 0.194-0.966) and LMFS (HR, 0.265; 95% CI, 0.078-0.900) than did the low CD68TF_Hotspot _group. Liver metastasis (HR, 8.144; 95% CI, 3.276-20.250) was an independent prognostic factor for OS. Additionally, patients with left colon cancer were prone to have a longer OS, whereas pathologic classification was not associated with OS (Table 3).

**Table 3:** Multivariate analyses of factors associated with OS and LMFS

Variable	OS (n = 98)		LMFS (n = 98)	
	
	HR, (95% CI)	P value	HR, (95% CI)	P value
Gender (female vs. male)	1.954 (0.841-4.538)	0.119	0.333 (0.083-1.335)	0.121

Age (< 60 y vs. ≥ 60 y)	0.504 (0.227-1.116)	0.091	0.881 (0.267-2.906)	0.835

Invasive depth (T4 vs. T3)	1.941 (0.693-5.436)	0.207	0.846 (0.171-4.190)	0.838

Site of primary tumors (right vs. left)	2.184 (0.981-4.859)	0.056	1.009 (0.298-3.414)	0.989

Grade (G3 vs. G2 vs. G1)	1.224 (0.457-3.281)	0.688	1.616 (0.345-7.575)	0.543

Pathologic Classification (mucoid + signet ring vs. papillary + tubular)	2.364 (0.787-7.100)	0.125	0.537 (0.071-4.061)	0.547

Growth patterns (infiltrating vs. pushing)	0.700 (0.295-1.662)	0.419	2.650 (0.551-12.746)	0.224

CD68TF_Hotspot _group (high vs. low)	0.433 (0.194-0.966)	0.041*	0.265 (0.078-0.900)	0.033*

Liver metastasis (yes vs. no)	8.144 (3.276-20.250)	0.000**	NA	NA

Multivariate analysis, Cox proportional hazard regression model. Abbreviations: HR, hazard ratio; CI, confidence interval; NA, not assessment. *: p < 0.05; **: p < 0.001.

## Discussion

By analyzing the relationship between the density of TAMs and the potential of hepatic metastasis and survival, this study showed that a higher density of macrophages in the invasive front of colon cancer was associated with a higher 5-year survival rate. Most importantly, the CD68TF_Hotspot _was associated with both the incidence of hepatic metastasis and the interval between colon resection and the occurrence of hepatic metastasis.

In contrast to other solid tumors, such as breast cancer, most studies have shown that TAMs, especially IL-12-positive TAMs, inhibit the progression of colon cancers [36-39,41-44]. For example, in Forssell's study [36] the higher macrophage infiltration along the tumor front correlated with improved survival in colon cancer compared to rectal cancer. In the current study, the Cox model indicated that the CD68TF_Hotspot _was independently prognostic. A higher 5-year survival rate after radical resection occurred in patients with a higher macrophage infiltration in the invasive front (81.0%) than in those with a lower macrophage infiltration (48.6%), which is in agreement with the previous studies [36-39].

The mechanisms behind the antitumor effects of TAMs have not been fully elucidated and could potentially be ascribed to the M1 phenotype, which is in part controlled by the CD4+T cells and the death of cancer cells [45-47]. TAMs with the M1 phenotype are characterized by a high capacity to present antigen, high IL-12 and IL-23 production, and high production of toxic intermediates, such as nitric oxide and reactive oxygen intermediates. Thus, TAMs with the M1 phenotype are generally considered potent effector cells which kill tumor cells [48-51]. In fact, TAMs showed a spectrum from M1 to M2 phenotypes in murine colon adenocarcinoma tumors [52]. This study showed that TAMs expressed with HLA-DR and IL-10 rather than TGF-β1 and IL-12, consistent with the previous observation [52]. Although an abundance of evidence relevant to the molecular mechanisms underlying the anti-tumor effect of macrophages has been documented, it is still unknown how TAMs exert a protective effect, except that one recent study indicated that TAMs reduce the development of peritoneal colorectal carcinoma metastases [36-39,41-44,53]. The current study analyzed the relationship between the infiltration of TAMs and hepatic metastasis. The results showed that a higher density of TAMs in the invasive front was associated with lower synchronous and metachronous hepatic metastases. Since hepatic metastasis of colon cancer is a key prognostic factor, this study might partly explain the reason that macrophage infiltration improves the prognosis of patients with colon cancer.

The molecular mechanisms underlying hepatic metastasis of colon cancers is poorly understood. Traditional clinicopathologic indices for hepatic metastasis of colorectal cancer, which include the depth of invasion, the presence of venous invasion, and lymph node metastasis, have only limited prognostic value [54]. Although multiple markers, such as CD10, CD44, VEGF, TGF-α, have been shown to be correlated with hepatic metastasis, the predictive efficacy of these markers is still unclear [55-60]. In the current study, a higher density of TAMs in the invasive front was associated with lower synchronous hepatic metastasis and lower metachronous hepatic metastasis, showing that the immune microenvironment of the primary tumor modifies the metastatic potential of colon cancer, and the function of TAMs is changeable in different tumor microenvironment [61].

Most immune cells, such as CD45RO+T cells, CD3+T cells, NK cells, TAMs, and even Treg cells, have shown a protective effect when infiltrated into colon cancer tissue [62-67]. Additionally, an autoimmune response is associated with the efficacy of biochemotherapy (GOLFIG regimen) for colon cancer [68,69]. The current study has given additional evidence that macrophage infiltration is involved in the inhibition of hepatic metastasis. These data indicate that colon cancer is an immunogenic tumor. Therefore, more attention should be paid to exploiting the immune response in an effort to improve conventional therapy for colon cancer [70].

Additionally, our study main aim is to find if there any relationship between macrophages and liver metastasis in colon cancer which was cut into the following three patterns: no hepatic metastasis, metachronous and synchronous liver metastasis. We decided to choose single stage IIIB colon cancer which is the biggest group in our center colon resource database to avoid the influence of different stages factor on relationship between macrophages and liver metastasis. Although this constitution minimized confounding factors, it cannot completely represent ordinary setup, so our results, and as such, should be viewed with some caution.

## Conclusion

This study demonstrated that TAMs infiltrated in the invasive front are associated with improvement in both hepatic metastasis and OS in colon cancer, implying that TAMs have protective potential in colon cancers and might serve as a novel therapeutic target.

## Competing interests

The authors declare that they have no competing interests.

## Authors' contributions

WXJ, DY, ZQM, PZZ, and WDS carried out the case collection; ZQ, XQ, and HJH carried out the immunohistochemical staining; and PRQ and ZX analyzed the results. ZXS and ZYX conceived the study, participated in the design, and coordinated and helped draft the manuscript. All authors read and approved the final manuscript.
